# Evaluation of Rare Earth Element-Associated Hormetic Effects in Candidate Fertilizers and Livestock Feed Additives

**DOI:** 10.1007/s12011-022-03331-2

**Published:** 2022-06-17

**Authors:** Franca Tommasi, Philippe J. Thomas, Daniel M. Lyons, Giovanni Pagano, Rahime Oral, Antonietta Siciliano, Maria Toscanesi, Marco Guida, Marco Trifuoggi

**Affiliations:** 1 Department of Biology, “Aldo Moro” Bari University, I-70125 Bari, Italy; 2 Environment and Climate Change Canada, Science & Technology Branch, National Wildlife Research Center – Carleton University, Ottawa, ON K1A 0H3 Canada; 3 Center for Marine Research, Ruđer Bošković Institute, HR-52210 Rovinj, Croatia; 4 Department of Chemical Sciences, Federico II Naples University, via Cintia, I-80126 Naples, Italy; 5 Faculty of Fisheries, Ege University, TR-35100 Bornova, İzmir, Turkey; 6 Department of Biology, Federico II Naples University, I-80126 Naples, Italy

**Keywords:** Rare earth elements, Hormesis, Toxicity, Fertilizer, Feed additive, Mixture

## Abstract

Rare earth elements (REEs) are recognized as emerging contaminants with implications in human and environmental health. Apart from their adverse effects, REEs have been reported as having positive effects when amended to fertilizers and livestock feed additives, thus suggesting a hormetic trend, implying a concentration-related shift from stimulation to inhibition and toxicity, with analogous trends that have been assessed for a number of xenobiotics. In view of optimizing the success of REE mixtures in stimulating crop yield and/or livestock growth or egg production, one should foresee the comparative concentration-related effects of individual REEs (e.g., Ce and La) vs. their mixtures, which may display distinct trends. The results might prompt further explorations on the use of REE mixtures vs. single REEs aimed at optimizing the preparation of fertilizers and feed additives, in view of the potential recognition of their use in agronomy and zootechny.

## Introduction

Rare earth elements (REEs) are a group of metals encompassing lanthanoids from lanthanum to lutetium, as well as yttrium and scandium, that have become indispensable in present-day life because of their critical role in many modern and cutting-edge technologies [1, 2]. In recent decades, an extensive body of literature on REE-associated adverse effects in a number of biota and laboratory test models has given cause for concern that environmental REE exposures may have deleterious impacts on flora and fauna [3]. A growing body of literature on human REE exposures in mining areas, including facilities dedicated to REE extraction and manufacturing, increasingly points to REE bioaccumulation and excretion. These include environmental, non-occupational exposures among residents in REE mining areas [4, 5], and point to the still many knowledge gaps on potential health risks in REE-exposed workers [6, 7].

Apart from industrial applications, REEs have been extensively used in Chinese agriculture as fertilizers to increase crop yield, and in zootechny as feed additives aimed at increasing livestock growth and egg laying, with likely prospects of their utilization outside China [7–10].

The REE-associated adverse effects and their stimulatory actions in plant and animal growth may be regarded as one more case of the hormesis phenomenon, as reviewed by Calabrese [11] and by Calabrese and Agathokleous [12].

In view of a possible hormetic trend for REEs, just as for an extensive number of agents already reported in the literature, it is increasingly clear that testing the dose–response trends of individual REEs as well as their combinations is of growing importance to identify the concentration ranges and combinations which can give rise to hormetic or toxic effects [13]. Resolving the doses at which hormesis may occur, as well as the nature of the hormetic effects, are discussed in the present review, with a special focus on the present state of art, as yet confined to Chinese agriculture and zootechny and on the possible extension of REE utilization in the production of fertilizers and feed additives in other countries, by appropriate authorization from food safety agencies.

## Materials and Methods

A detailed reference search of the literature was carried out using the PubMed, Scopus, and ScienceDirect databases by interfacing the following keywords:

1. Rare earth elements vs. hormesis; vs. toxicity; vs. fertilizer; vs. feed additive, and vs. mixture
2. Hormesis vs. metal, and vs. mixture

No data from human REE exposures are reported in the present review.

## REE-Associated Adverse Effects

After the pioneering studies by Drobkov [14] in 1941 on the effects of REEs on the development of peas, and by Jha and Singh [15, 16] assessing the induction of cytogenetic damage by two REEs (praseodymium and neodymium) in mice and in broad bean (*Vicia faba*), a thriving literature over recent decades has provided established evidence for a number of REE-associated adverse effects in a number of test models, as summarized in Table 1. Studies of REE toxicity in plant models were carried out on several crop and native species, showing decreased seed germination, root elongation, and mitotic activity for REE levels ≤ 5.0 mg/L [17–22]. More extensive studies of REE-associated toxicity were conducted in several animal models including mammals (mice and rats), fish (*Danio rerio*), and sea urchins, providing evidence for a number of adverse effects, including oxidative damage, lung and kidney toxicity, and developmental and cytogenetic damage [23–37]. Altogether, the available body of literature on the adverse effects of REE exposures raises environmental health concerns.

**Table 1:** Selected REE-related literature: adverse effects

Test models	Test REEs [concentration]	Endpoints	Observed effects	References
Plants				
* Triticum aestivum*	La and Ce [0.5–25 mg/L]	Root elongation; dry weight of roots and shoots; content of mineral elements	Decreased parameters	Hu et al. [17]
* Brassica juncea* var. *crispifolia*	La[III] [0.05–5.0 mg/L]	Root elongation; Fe, Mn, and Zn accumulation	La [≥ 1.0 mg/L] inhibited root elongation and metal accumulation	Xiong et al. [18]
5 native and crop plants	La, Ce, and Y [20–2000 mg/kg]	Germination and harvest	Decreased germination	Thomas et al. [19]
6 native and crop plants	Pr, Nd, Sa, Tb, Dy, Er [100–700 mg/kg]	Seed germination; speed of germination	Decreased germination	Carpenter et al. [20]
* Allium cepa*	La and Ce [0–200 mg/L]	Root growth; mitotic index and frequency of aberrant cells	Decreased growth; mitotic index and increased aberrant cells	Kotelnikova et al. [21]
* Raphidocelis subcapitata*	La and Ce [0–0.5 mg/L]	Growth inhibition; superoxide dismutase, catalase	Decreased growth, increased oxidative stress	Siciliano et al. [22]
Animals				
Mice [adult and fetal]	CeCl_3_ [gavage] [200 or 500 mg/kg BW]	Pulmonary hemorrhage [adults], pulmonary and hepatic vascular congestion [neonatal]	Increased pulmonary damage	Kawagoe et al. [23]
Wistar rats	LaCl_3_ [gavage] [0.1–40 mg/kg]	Behavioral performance; [Ca^2+^]_i_ level; Ca^2+^_i_-ATPase in hippocampal cells; oxidative stress	Increased Ca^2+^_i_-ATPase; decreased activities of antioxidant enzymes	He et al. [24]
Rats	CeO_2_ [nanoparticles] [175–250 mg/kg]	Oxidative stress endpoints	Increased oxidative stress in cortex, hippocampus, and cerebellum	Hardas et al. [25]
Mice	La, Ce, and Nd[III] [by gavage]; [10, 20, or 40 mg/kg BW/day] 6 weeks	Accumulation in hepatocyte, nuclei, and mitochondria	Oxidative damage in hepatic nuclei and mitochondria	Huang et al. [26]
Sprague–Dawley rats	CeO_2_ nanoparticles [1.0–7.0 mg/kg]	Liver ceria levels; serum alanine transaminase; albumin levels	Decreased liver weight; hydropic degeneration; hepatocyte	Nalabotu et al. [27]
ICR mice	LaCl_3_, CeCl_3_, and NdCl_3_ [20 mg/kg BW, i.p.]	Brain injury; oxidative stress	Increased brain injury and oxidative stress	Zhao et al. [28]
ICR mice	CeCl_3_ [gavage] [2–20 mg/kg BW]	Hepatocyte ultrastructure; oxidative stress; kidney structure	Increased ROS formation; inhibited stress-related gene expression	Zhao et al. [29, 30]
CD1 Mice	CeO_2_ nanoparticles [2 mg/m^3^]	Pro-inflammatory cytokines; oxidative stress markers	Increased pro-inflammatory condition	Aalapati et al. [31]
Mice	CeCl_3_ [2 mg/kg] via gavage	Liver injury and gene-expressed profiles	Decreased counts of white blood cells; lymphocytes; platelets; reticulocyte count; neutrophilic granulocyte percentages; A/G ratio	Cheng et al. [32]
Mice	CeCl_3_ [nasally instilled]	Pro-inflammatory lung parameters; serum triglyceride levels	Oxidative stress and inflammatory cytokine expression; sinusoidal dilatation	Hong et al. [33]
* Caenorhabditis elegans*	La^3+^ [10 μM]	Growth and reproduction	Significant adverse effects	Zhang et al. [34]
Zebrafish embryos	La^3+^ or Yb^3+^ [0.01 to 1 mM]	Developmental defects and mortality	Increased damage	Cui et al. [35]
3 sea urchin species [embryos and sperm]	7 REE chlorides [10^−6^–10^−4^ M]	Developmental defects; fertilization success; offspring anomalies; cytogenetic damage	Increased developmental defects; decreased fertilization; increased cytogenetic anomalies	Oral et al. [36]; Trifuoggi et al. [37]

## REE-Associated Hormetic Trends

Analogous to a number of chemical and physical agents [11, 38], REE dose–response trends have been associated with hormesis, a phenomenon leading to stimulate (Greek: hormào) biological activities at lower concentrations compared to inhibition, bioaccumulation, and toxicity at higher exposure concentrations [39]. As shown in Table 2, evidence for REE-associated hormetic trends were reported in a set of studies conducted in several biota including plants, fungi, microbiota, and animals.

**Table 2:** Hormetic effects in growth endpoints

Test models	Test REEs [concentration]	Endpoints	Observed effects	References
Plants				
Rice [*Oryza sativa*]	La[NO_3_]_3_ [20–1500 μg/mL]	Germination of rice seeds; chlorophyll contents; root growth	Increased parameters	Fashui et al. [40]
Broad bean [*Vicia faba*]	LaCl_3_ [108–195 μg/g]	Superoxide dismutase; catalase; ascorbate peroxidase; HSP 70	Hormetic effects	Wang et al. [41]
Chinese cabbage [*Brassica rapa*]	LaCl_3_ and CeCl_4_	Soluble sugar, titratable acid, nitrate and vitamin C	La more effective than Ce; different data for autumn vs. spring plantation	Ma et al. [42]
Soybean [*Glycine max*]	La[III] [5–150 μM]	Growth; mitotic index; chlorophyll content	Low La concentrations stimulated, high concentrations decreased the photosynthetic rate	de Oliveira et al. [43]
Rice [*Oryza sativa*]	La[III] [0.05–1.5 mM]	Redox endpoints	Increased catalase and peroxidase by 0.05 and 0.1 mM La[III]	Liu et al. [44]
* Capsicum annuum*	LaCl_3_ [10 μM]	Seedling height; shoot diameter	Increased growth	García-Jiménez et al. [45]
Rice[*Oryza sativa*]	Sc[III] [25 and 50 μM]	Germination; oxidative stress parameters	Improved germination; decreased oxidative stress	Elbasan et al. [46]
* Phaseolus vulgaris*	Ce[NO_3_]_3_ 6H_2_O [0.1–72.9 mM]	Survival rate and growth vs. water stress	Increased photosynthesis rate, chlorophyll content, and water use efficiency	Salgado et al. [47]
Orange [*Poncirus trifoliate*]	Ce[NO_3_]_3_ 6H_2_O [0.25–4 mM]	Growth kinetics; chlorophyll content	Different hormetic effects	Yin et al. [48]
Fungi and microbes				
* Trichoderma atroviride* and *Trichoderma harzianum*	La and REE mix [0.003 to 900 mM]	Accumulation of REE in fungal biomass	Increased growth	d’Aquino et al. [49]
* Escherichia coli*	16 REEs	Growth kinetics	Different hormetic effects	Técher et al. [50]
Microbial communities	Y[III] [≤ 20 mg/L] or [20–500 mg/L]	Ammonia-oxidizing bacteria	Increased specific oxygen uptake rate at ≤ 20 mg/L; decreased > 20 mg/L	Su et al. [51]
Animals and animal cells				
Human dermal fibroblasts	14 REE ions [1–100 μM]	Pro-fibrotic responses in tissue injury	Increased proliferation by low REE levels; decreased proliferation by higher REE levels	Jenkins et al. [52]
Murine preosteoblast cell line MC3T3-E1	LaCl_3_ [10^−9^–10^−3^ M]	Proliferation; osteogenic differentiation, and mineralization	Upregulated below 10^−6^ M, downregulated at 10^−3^ M	Liu et al. [86]
Mice	CeO_2_ [nanoparticles] [0.5 mg/kg]	ROS production	Decreased ROS	Hirst et al. [53]
Sprague–Dawley rats	Y_2_O_3_ [20–320 ppm]	Body weight; spatial learning and memory; anogenital distance	Increased at 20 ppm; decreased at 320 ppm	Zhang et al. [54]

In particular, plant models including rice, bean, cabbage, and orange were exposed to varying levels of La, Ce, and Sc by testing some key endpoints including growth, germination, chlorophyll content, and oxidative stress parameters. The results reported on concentration-related hormetic trends in REE-exposed plants [40–48]. de Oliveira et al. [43] tested La^3+^ exposures (5 to 150 μM) in soybean plants, by measuring a set of endpoints at low REE concentrations as plant growth, nutritional characteristics, photosynthetic rate, chlorophyll content, mitotic index, modifications in the ultrastructure of roots and leaves, and La mapping in root and shoot tissues. When La was applied, it was noted that the levels of some essential nutrients (Ca, P, K, and Mn) increased. Low La concentrations enhanced the photosynthetic rate and total chlorophyll content and led to a higher incidence of binucleate cells, with a slight increase in root and shoot biomass. At higher La levels, soybean growth was reduced. Liu et al. [44] tested La^3+^ (0.05 to 1.5 mM) in rice plants for effects on reactive oxygen species and antioxidant metabolism. The results indicated that ROS levels declined after treatment with 0.05 mM La^3+^, with hormetic effects on the antioxidant metabolism in rice roots. Further, d’Aquino et al. [49] tested *Trichoderma* fungi to REE exposures ranging from 0.003 to 900 mM, and found increased growth of fungal biomass at low REE concentrations. Extending this work to bacteria, *E. coli* or microbial communities were exposed to several REEs by Técher et al. [50] and to Y(III) by Su et al. [51], who found increased growth kinetics and ammonia-oxidizing bacteria at low (< 20 mg/L) Y(III) concentrations but were inhibited by higher (≥ 20 mg/L) Y(III) concentrations.

Several studies of REE-associated hormetic effects were conducted in animal models (Table 2). Jenkins et al. [52] tested human dermal fibroblasts for profibrotic injury when exposed to REEs and found increased proliferation by low concentrations of REEs (1 to 10 μM), which turned to inhibition at higher (50 to 100 μM) REE concentrations. Decreased inflammatory parameters were reported by Hirst et al. [53] in mice exposed to low concentrations of CeO_2_ nanoparticles. More recently, Zhang et al. [54] tested the response of rats to Y_2_O_3_ exposure for growth endpoints, which were found to increase at low concentrations (20 ppm) and decrease at higher Y_2_O_3_ concentrations (320 ppm).

## REEs in Fertilizers

The established use of REEs as fertilizer components in Chinese agriculture dates back to the 1980s and was reported in early reviews [7, 55, 56]. A few reports in the past decade have focused on some molecular endpoints in plants exposed to REE-containing fertilizers. Xu and Wang [57] found increased phosphorus uptake in maize after application of REE (La and Ce)-containing fertilizer, with applications of less than 10 kg/ha reported as increasing crop yield. Cheng et al. [58] exposed navel orange (*Citrus sinensis*) plants to a REE mixture (38.6 to 546 mg/kg in soil) by measuring a set of fruit quality indicators, including titratable acidity, total soluble solids, and vitamin C. The outcome was improved internal fruit quality in REE-exposed navel orange. A recent report by Lian et al. [59] investigated the effects of La^3+^ on growth, photosynthetic ability, and phosphorus-use efficiency (PUE) in various organs of adzuki bean *Vigna angularis* seedlings. Treatment of young seedlings with La^3+^ at 150 mg/L improved PUE in roots, stems, and leaves via the regulation of root elongation and activation of root physiological responses to P deficiency. La^3+^ increased the level of superoxide dismutase and peroxidase, while it significantly decreased malondialdehyde content. The negative effects of P-deficiency on net photosynthetic rate, transpiration rate, and chlorophyll content in leaves were alleviated by La^3+^ treatment.

## REEs in Livestock Feed Additives

Analogous to their use in fertilizers, REEs have been used in Chinese zootechny as livestock feed additives, as reported by Wang and Xu [60] in their review of an extensive body of literature encompassing Chinese and Japanese papers dating back to the 1980s and the 1990s, and in a recent review by Abdelnour et al. [61]. Mechanistic and up-to-date reports are summarized in Table 3. He et al. [62] tested diet supplementation of a REE mixture in piglets (300 mg/kg) and reported an increased body weight gain and feed conversion ratio. The same positive effects were found by Wang and Xu [60] who supplemented piglets with LaCl_3_ (100 mg/kg BW). A recent study by Xiong et al. [63] evaluated the effects of a REE mixture (200 mg/kg BW) on sows and their offspring, observing improved antioxidant activity, immunity, reproduction of sows, and growth of piglets. Liu et al. [64] supplemented Simmental steers with LaCl_3_ (400 to 1800 mg/day) and found improved rumen fermentation, urinary excretion, and feed digestibility. Renner et al. [65] supplemented fattening bulls with a mixture of REE citrates (100 to 300 mg/kg dry matter) and found that REE supplementation affected dry matter intake, but not live weight gain, clinical chemical parameters, and ion concentrations significantly. Peripheral blood mononuclear cells were significantly increased in REE-supplemented bulls. He et al. [62] fed Ross broiler chicks with either the chloride or citrate salts of REEs, and found improved growth performance of broilers without affecting carcass composition and health of the broilers. Cai et al. [66] fed broiler chickens with REE-enriched yeast (500 to 1500 mg/kg BW) and found improved growth performance. Durmuş and Bölükbaşı [67] supplemented laying hens with La_2_O_3_ (100 to 400 mg/kg BW) and observed improved feed conversion ratio, egg production, and egg shell life. In further work, the same group [8] supplemented laying hens with CeO_2_, finding similar results as increased egg shell breaking strength and decreased oxidative stress parameters.

**Table 3:** Selected REE-related literature: use of REE-based feed additives

Animal groups	REE	Endpoints	Observed effects	References
Pigs				
[Duroc × Landrace × Yorkshire] piglets	LaCl_3_ [100 mg/kg BW]	Average daily weight gain; feed conversion ratio	Increased parameters	Wang and Xu [60]
Deutsche Landrasse × Piétrain piglets	REE mixture [300 mg/kg BW]	Body weight gain; feed conversion ratio	Improved endpoints	He et al. [61]
[Landrace × Yorkshire] × Duroc finishing pigs	REE-enriched yeast [500 – 1500 mg/kg BW]	Average daily weight gain; gain to feed ratio	Improved endpoints	Cai et al. [62]
Sows and offspring	REE mixture [200 mg/kg BW]	Antioxidant activity; immunity; reproduction of sows and piglets; growth of offspring; microbiota	Endpoints improvements	Xiong et al. [87]
Cattle				
Simmental steers	LaCl_3_ [400–1800 mg/day]	Rumen fermentation, urinary excretion, digestibility	Improved endpoints	Liu et al. [63]
Fattening bulls	REE citrate [100–300 mg/kg dry matter]	Dry matter intake; weight gain; chemical parameters	Contrasting outcomes	Renner et al. [64]
Fowl				
Ross broiler chicks	REE-chloride [40 mg/kg] REE-citrate [70 mg/kg]	Weight increase [chill, breast, wing]	Improved growth performance	He et al. [65]
ROSS 308 broilers	REE- enriched yeast [500–1500 mg/kg BW]	Gross energy digestibility; growth performance, and relative organ weight	Improved endpoints	Cai et al. [88]
Lohman LSL hens	La_2_O_3_ [100–400 mg/kg BW]	Egg quality, fatty acids composition of yolk, and egg lipid peroxidation	Improved feed conversion ratio; egg production, and egg shell life	Durmuş and Bölükbaşı [66]
Lohman LSL hens	CeO_2_ [100–400 mg/kg BW]	Feed conversion ratio and egg production	Increased egg shell breaking strength; decreased oxidative stress	Bölükbaşı et al. [8]

Beyond those experimental reports, it must be recognized that an official stamp of approval for the use of REE-based feed additives in a more widespread way globally is yet to be forthcoming, as reviewed by Squadrone et al. [68]. At least in one case, to our knowledge, a safety statement was provided by the EFSA Panel FEEDAP [69] for the feed additive Lancer®, a REE citrate mixture to be used in piglet diet. The EFSA Panel stated that uncertainty still remains on possible developmental neurotoxicity of Lancer® since it was unable to identify a no observed adverse effect level. However, the FEEDAP Panel considered that exposure to La and Ce from products of animals treated with Lancer® at 250 mg/kg feed would not add a significant contribution to the background exposure of these elements. The FEEDAP Panel concluded that the use of Lancer® in feed for weaned piglets according to the proposed conditions of use does not represent a safety concern for the consumer and for the environment.

Though there is currently little data available on the progress of other candidate feed additives, it is to be expected that increasing knowledge on the hormetic effects of REE-based materials will lead to further regulatory approval of REE-containing feed additives in the not-too-distant future.

## Toward Production of REE Mixtures as Hormetic Agents

Under a historical perspective, the pioneering studies of hormesis by Stebbing [70] in 1982, revisiting the nineteenth-century Arndt-Schulz Law, have now made hormesis a well-known phenomenon in biological sciences, medicine, and pharmacology. In the more specific fields of agriculture and zootechny, and in the use of REEs as ingredients for fertilizers and feed additives, a persuasive body of evidence reports advantages to using REEs for increasing crop yield and livestock performance. Indeed, as well-theorized by Edward Calabrese and his group [11–13, 38], REEs display hormetic dose–response trends, just as with a number of other chemical and physical agents, which are being underpinned with increasingly sophisticated theoretical frameworks [71–74]. However, it should be noted that REEs are rarely present individually but usually more likely as a mixture of REEs. For this reason, it is timely to begin considering the effect on biota of multiple REEs concomitantly present, particularly at very low concentrations and how hormetic effects might be modulated or negated. For example, as observed by Jacob et al. [75], when pharmaceuticals such as diazepam and simvastatin are individually present at concentrations below the no observable effect concentration, combinations of these at such concentrations indicate toxicity, e.g., to *Aliivibrio fischeri*. Hence, the need should be recognized for more studies involving mixtures, particularly at very low concentrations, since chemicals are subject to interactions and modifications which may result in antagonistic, additive, or synergistic effects.

This was the case, reported in our early studies [76, 77], of a shift from stimulation to inhibition of sea urchin sperm fertilization rate by exposures to sub-micromolar levels of either cadmium or zinc, compared to their mixtures, respectively. Subsequent and recent investigations have further explored the concentration-related hormetic trends of several agents compared to their binary or multiple mixtures, such as antibiotics [78–80], industry wastewater [81], pharmaceuticals [82–84], and fungicides [85].

In view of likely developments in the production and use of REE-based fertilizers and feed additives, and in view of open questions persisting on the efficacy of using REE mixtures and their concentration-related trends, ad hoc investigations are required aimed at verifying the single vs. combined use of REEs in these production and use scenarios.
